# Predictors of asthma‐related quality of life in a large cohort of asthmatics: A cross‐sectional study in a secondary care center

**DOI:** 10.1002/clt2.12054

**Published:** 2021-09-03

**Authors:** Gilles Louis, Benoit Pétré, Florence Schleich, Halehsadat Nekoee Zahraei, Anne‐Françoise Donneau, Aude Silvestre, Monique Henket, Virginie Paulus, Françoise Guissard, Michèle Guillaume, Renaud Louis

**Affiliations:** ^1^ Department of Public Health University of Liège Liège Belgium; ^2^ Department of Pneumology GIGAI^3^ University of Liège Liège Belgium; ^3^ Department of Public Health Biostatistics Unit University of Liège Liège Belgium

**Keywords:** asthma control, asthma‐related quality of life, FeNO, Asthme, Contrôle, qualité de vie liée à l'asthme, NO exhalé

## Abstract

**Background:**

In recent decades, asthma‐related quality of life questionnaires have joined objective clinical indicators as important outcome measures. In this study, we sought to investigate the predictors of asthma‐related quality of life in a large cohort of patients recruited from a secondary care center.

**Methods:**

We conducted a cross‐sectional study on asthmatics (*N* = 1301) recruited from the Liège University Hospital asthma clinic (Belgium). After performing a descriptive analysis highlighting the distribution of scores from the Mini Asthma Quality of Life Questionnaire (Mini AQLQ) and its four dimensions (symptoms, activity limitation, emotional function, and environmental stimuli), we did multiple regression analysis to identify the independent predictors of AQLQ.

**Results:**

Multiple regression beta analysis showed that AQLQ and its four dimensions were primarily associated with asthma control (*p* < 0.0001 in all instances). Female gender was associated with a lower score for the AQLQ's activity and environmental dimensions (*p* < 0.05 for both), while current smokers had a higher score on the AQLQ's environmental dimension (*p* < 0.0001). The burden of asthma treatment was associated with a lower score for the AQLQ's emotional (*p* < 0.05) and environmental (*p* < 0.05) dimensions. BMI was associated with a lower score in the AQLQ's activity dimension (*p* < 0.0001), while the opposite was true for the FeNO test (*p* < 0.0001). Sputum neutrophils were inversely related to the score for the AQLQ's symptom dimension (*p* < 0.05), whereas post‐bronchodilator FEV_1_ showed a positive relationship for that same dimension (*p* < 0.05).

**Conclusion:**

Asthma control is the main predictor of AQLQ score and impacts all its dimensions, but demographic, functional, and airway inflammatory parameters may also influence some dimensions of the AQLQ.

## 1 INTRODUCTION

Asthma is defined by the Global Initiative of Asthma (GINA) as a heterogeneous disease, usually characterized by chronic airway inflammation. It is diagnosed by a history of respiratory symptoms such as wheezing, shortness of breath, chest tightness, and cough that vary over time and in intensity, together with variable expiratory airflow limitation.^1^

This disease is a growing burden in terms of morbidity, health care costs, and health‐related quality of life (HRQL).^2^ In recent decades, as a result of a paradigm shift towards patient‐centered care, subjective measures of HRQL in asthma have become important outcomes alongside objective clinical outcomes.^3^ International asthma treatment guidelines have therefore evolved to include improvement in patients' HRQL through long‐term control of the disease, minimizing symptoms, and improving physical, psychological, and social functioning.^4^

Unravelling the predictors of asthma‐related quality of life is important for understanding the disease and its treatment, and should provide meaningful information about the impact of the disease and its treatment on patients' perceived health.^5^

Many previous studies have explored factors associated with asthma‐related quality of life.^6^ They showed that the main factors were asthma control,^7^, ^8^, ^9^ gender,^10^, ^11^, ^12^, ^13^ age,^9^, ^13^ body mass index (BMI),^12^, ^14^ education,^9^ and sociodemographic parameters.^11^, ^13^ Some studies have also shown a weak univariate relationship between HRQL and lung function as measured by forced expiratory volume in 1 s (FEV_1_).^13^, ^15^

As for the inflammatory component, some studies have explored the relationship between asthma‐related quality of life and the fraction of exhaled nitric oxide (FeNO), with contrasting results.^16^, ^17^, ^18^ To the best of our knowledge, no studies have explored the impact of granulocytic sputum cell count on asthma‐related quality of life, although airway inflammation is included in the definition of asthma. In this study we assessed the predictors of asthma‐related quality of life in a large cohort of asthmatics who were well characterized with respect to demographic features, lung function, and systemic and airway inflammatory parameters.

## 2 METHODS

### 2.1 Study design, setting, and participants

**FIGURE 1:**
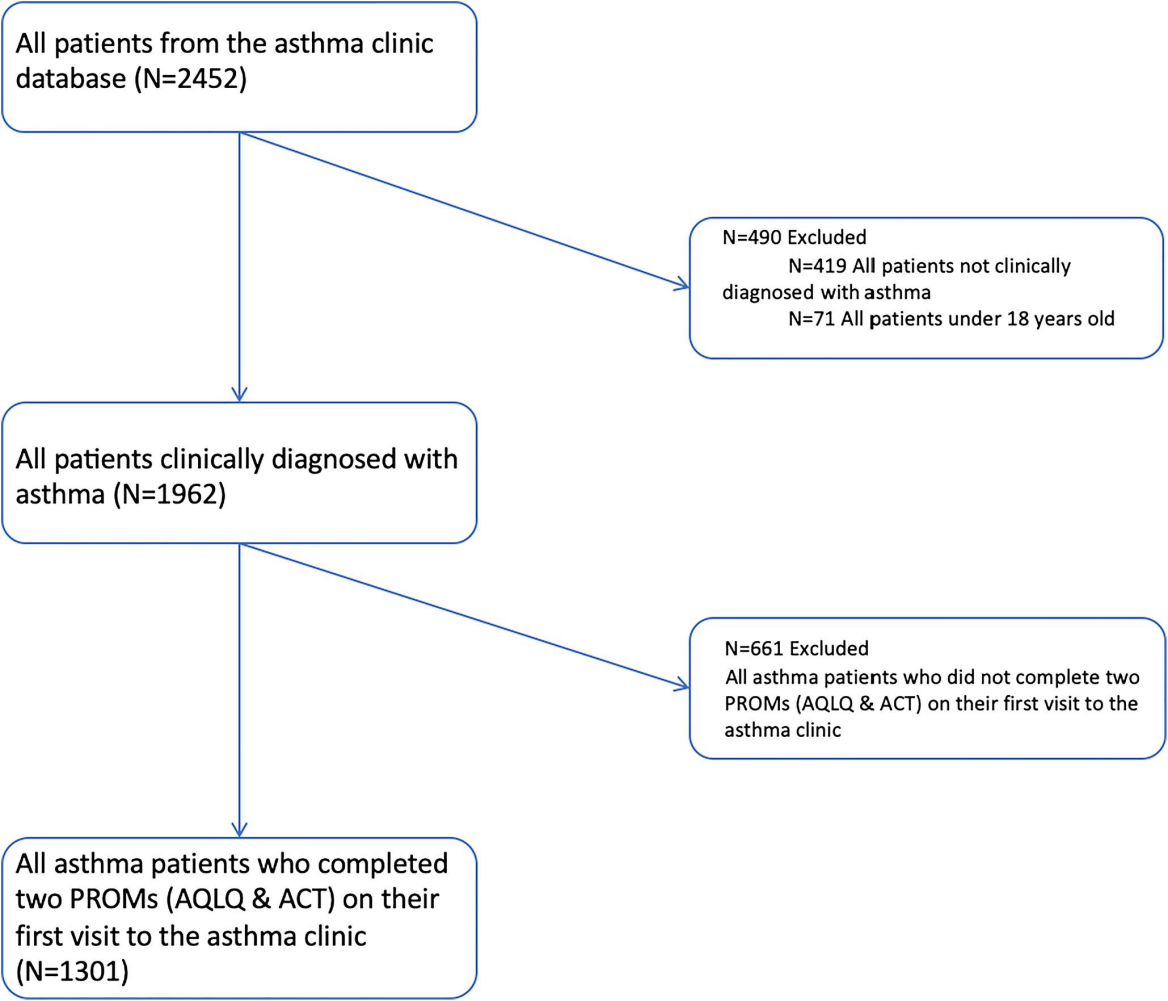
Flow chart of the patient selection process. ACT, Asthma Control Test; AQLQ, Asthma Quality of Life Questionnaire; PROM, patient‐reported outcome measure

This was a cross‐sectional study on asthma patients recruited from the Liege University Hospital Asthma Clinic (Belgium) between 2003 and 2019. In accordance with the GINA, the asthma diagnosis was based on the presence of typical symptoms (wheezing, breathlessness, chest tightness, and cough) combined with an FEV_1_ that increased at least 12% over baseline and was at least 200 ml after inhalation of 400 μg salbutamol and/or a provocative concentration of methacholine causing a 20% drop in FEV_1_ ≤16 mg/ml. We excluded patients under the age of 18 years and those not clinically diagnosed with asthma who failed to meet the reversibility or bronchial hyperresponsiveness criteria. Then, from the patients with asthma, we selected those who completed two asthma‐related patient‐reported outcome measures (PROMs), namely the Asthma Control Test (ACT)^19^ and the Mini Asthma Quality of Life Questionnaire (Mini AQLQ)^20^ on their first visit to the asthma clinic. The sample size was 1301 asthmatics (Figure 1).

### 2.2 Variables

All the variables used in this study were entered into the asthma clinic database during routine visits.

#### 2.2.1 Asthma‐related quality of life (dependent variable)

Asthma‐related quality of life was measured using the Mini Asthma Quality of Life Questionnaire (Mini AQLQ). This scientifically‐validated questionnaire has been translated into more than 20 languages and is intended for adults with asthma. It includes 15 items divided into four different dimensions: symptoms (5 items), activity limitation (4 items), emotional function (3 items) and environmental stimuli (3 items). The 15 items are scored on a seven‐point Likert scale; the score for the questionnaire as a whole and for the individual dimensions are simply averages of the responses to the questions within them.^20^ Seven is the highest score in terms of asthma‐related quality of life. The minimal clinically important difference—the smallest difference in a quality of life score that the patient perceives as clinically important^21^—is 0.5 for Mini AQLQ.

#### 2.2.2 Independent variables

We selected different variables from our database that might influence asthma‐related quality of life. We gathered them into two main groups: patient demographic characteristics and disease characteristics.

Patient demographic characteristics were age, gender, BMI, smoking status, age of onset of asthma, and treatment. Smoking status was divided in three categories: never‐smoker, ex‐smoker (quit smoking at least 6 months previously) and current smoker. Treatment was divided in four categories: (1) no treatment; (2) SABA alone as needed; (3) maintenance treatment including ICS + LABA and/or LTRA and/or LAMA; (4) any maintenance treatment and oral corticosteroid (OCS). Exacerbations in the year prior to the visit was defined by at least a three‐day course of OCS in non‐OCS treated patients and a quadrupling in dose for patients on maintenance OCS.

Disease characteristics were asthma control, atopy, lung function, and systemic and airway inflammation. Asthma control was assessed using the Asthma Control Test (ACT), which consists of five questions related to symptomatology and activity limitation. Each question contains five items, each scored on a scale from 1 to 5. Studies have established cutoff scores for asthma that is well‐controlled (ACT ≥ 20), not well‐controlled (ACT ≤ 19), and uncontrolled (ACT ≤ 15).^19^, ^22^ Atopy was defined by one positive IgE test (>0.35 kU/L) to one or more common aeroallergens (grass pollen, tree pollen, cat, dog, molds, and house dust mite). Lung function testing was performed by spirometry (Spiro bank; MIR, Rome, Italy). A post‐bronchodilator (reversibility) test was done for each patient, irrespective of their baseline FEV_1_ and FEV_1_/forced vital capacity (FVC) ratio, as a standard procedure. The best of three consecutive spirometry readings was used, as recommended by the European Respiratory Society. Patients were administered 400 μg of inhaled salbutamol via a metered‐dose inhaler (Ventolin) and a spacer (Volumatic), one puff at a time into the spacer, and spirometry was performed again 15 min later.^23^ Patients with baseline FEV_1_ ≥ 70% predicted were given a methacholine challenge test, as previously described.^23^ Using tidal breathing, the subjects inhaled successive quadrupling methacholine concentrations from 0.06 mg/ml to 16 mg/ml for one minute each; FEV_1_ was measured 30 and 90 s after each concentration. The test was stopped if FEV_1_ fell at least 20% from its baseline value. The PC20M was calculated by linear interpolation from the last two points of the curve. Inflammatory parameters included fractional exhaled nitric oxide (FeNO), sputum cell counts, blood cell counts, and systemic markers. FeNO was measured at a flow rate of 50 ml/s (NIOX®; Aerocrine, Solna, Sweden). Sputum induction and processing were performed as previously described.^24^ The success rate of sputum induction and analysis in our asthma clinic was 78% (1013 of 1301 asthma patients). C‐reactive protein (CRP), fibrinogen, blood eosinophils, and neutrophil counts were determined by routine laboratory analysis at Liège University Hospital.

### 2.3 Statistical analysis

The normality of the distribution of the quantitative data was evaluated numerically by comparing mean and median and graphically using a histogram and quantile‐quantile plot. The Shapiro‐Wilk test for normality was used to complete this assessment. Quantitative variables were summarized accordingly using median and interquartile range (P25–P75), while counts and percentages were calculated for qualitative variables. The number and percentage of missing values were also reported. The associations between the quantitative variables and the AQLQ and its subscales (symptom dimension, activity dimension, emotional dimension, and environmental dimension) were first determined using the Spearman correlation coefficient. We further analyzed the determinants of AQLQ and its four subscales by multiple regression analysis. Based on the skewed and bounded—that is, values restricted to the interval between 1 and 7 – structure of the outcome variable, a beta regression model was considered. To do that, the outcome values were rescaled to the unit interval using the following transformation y^* = ([y (n‐1)+0.5])/*n* where *n* is the sample size.^25^ In this study, the best of the several models fitted was selected using the Akaike information criterion (AIC), with a lower AIC value indicating a better fit. All statistical modeling was done using R statistical software at a significance level of 0.05.

### 2.4 Ethics

This study was approved by the Liège University Hospital ethics committee. Signed informed consent was obtained from patients as soon as they entered the asthma clinic. They agreed to allow their clinical data and the health outcomes they reported in the routine setting to be used for research purposes.

## 3 RESULTS

### 3.1 Characteristics of the study population

The demographic characteristics of the study population are presented in Table 1. The majority of subjects were female (60%). Never‐smokers represented 54% of our population, while ex‐smokers and current smokers accounted for 26% and 20% of the population, respectively. Atopy was found in a slim majority of patients (54%). The median BMI was 26 kg/m^2^, with an interquartile range (IQR) from 23–30, meaning that 25% of our population was obese. The vast majority of asthma patients were receiving medical treatment for asthma at the time of the visit (84%); the types of treatment are detailed in Table 1. Thirty‐seven percent of patients (440/1195) reported an exacerbation in the 12 months prior to the visit.

**TABLE 1:** Demographic characteristics

Variable		Median (IQR)/Percentage (*n*)	Percentage (number) of missing values
Age (years)		51 (37–62)	0% (0)
Gender (male)		40% (523)	0% (0)
Smoking status	Non‐smoker	54% (698)	0.69% (9)
	Ex‐smoker	26% (340)	
	Smoker	20% (254)	
Age of onset of asthma (years)		22 (10–50)	21% (270)
BMI (kg/m^2^)		26 (23–30)	0.08% (1)
Atopy (yes)		54% (648)	9% (113)
Exacerbations (yes)		37% (440)	8% (106)
Any asthma treatment (yes)		84% (1095)	0.2% (2)
No treatment		15.7% (204)	0% (0)
SABA alone as needed		22.4% (291)	0% (0)
Maintenance treatment (ICS/LABA and/or LTRA and/or LAMA)		51.6% (671)	0% (0)
Any maintenance treatment + OCS		10.2% (133)	0.2% (2)

Abbreviations: BMI, Body Mass Index; ICS, Inhaled corticosteroids; IQR, interquartile range; LABA, long‐acting beta agonists; LAMA, long‐acting antimuscarinics; LTRA, leukotriene receptor antagonists; n, number of patients; OCS, oral corticosteroids; SABA, short acting beta agonists.

**FIGURE 2:**
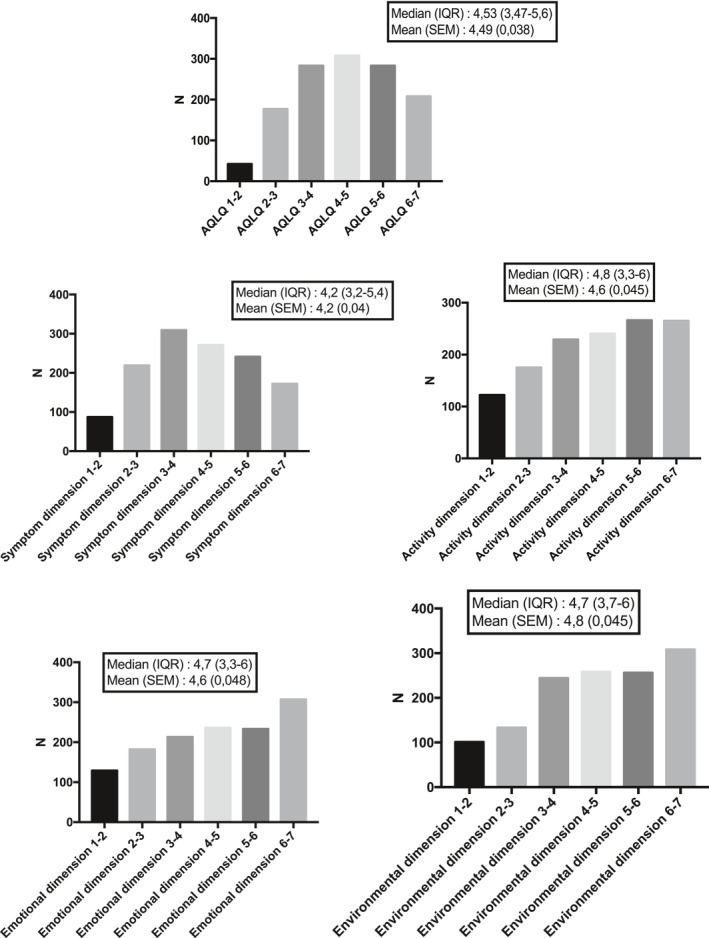
Distribution of the global AQLQ score and its four dimensions for the entire cohort (*N* = 1301). AQLQ, Asthma Quality of Life Questionnaire; IQR, interquartile range; SEM, standard error of the mean

The median AQLQ score was 4.53 with a non‐Gaussian distribution, as shown in Figure 2. Likewise, the distributions of the different AQLQ dimensions were nonparametric (Figure 2). Asthma control, lung function, and inflammatory characteristics are shown in Table 2. For asthma control, the median ACT was 15 with an IQR of 11–20, indicating that 25% of the cohort had well‐controlled asthma (ACT ≥ 20).

**TABLE 2:** Asthma control, lung function, and inflammatory characteristics

Variable	Median (IQR)/Percentage (frequency)	Percentage (number) of missing value
ACT	15 (11.0–20.0)	0% (0)
FEV_1_ pre (% pred.)	86 (73.0–99.0)	0.23% (3)
FEV_1_ post (% pred.)	91 (78.5–104.0)	0.61% (8)
FVC pre (% pred.)	95 (82.0–106.0)	0.15% (2)
FVC post (% pred.)	97 (85.0–108.0)	0.69% (9)
FEV_1_/FVC pre (%)	76 (69.0–82.0)	0.15% (2)
FEV_1_/FVC post (%)	79 (72.0–84.0)	0.61% (8)
Reversibility (%)	5.0 (1.0–11.0)	0.5% (7)
PC20M (mg/ml)	3.5 (0.92–14.21)	40.5% (527)
FeNO (ppb)	22 (13.0–42.0)	2.07% (27)
Sputum neutrophils (10^3^/g)	764 (272–2662.5)	22.14% (288)
Sputum eosinophils (10^3^/g)	27 (1.12–192.66)	22.14% (288)
Blood neutrophils (1/μl)	3966 (3077–5254)	2.0% (26)
Blood eosinophils (1/μl)	171 (98.0–298.0)	2.0% (26)
Total serum IgE (kU/L)	98 (30.0–291.0)	4.15% (54)
Fibrinogen (g/L)	3.25 (2.75–3.78)	5.69% (74)
CRP (mg/L)	2.15 (0.9–5.01)	4.84% (63)

Abbreviations: ACT, Asthma Control Test; BMI, Body Mass Index; CRP, C‐reactive protein; FENO, fraction of exhaled nitric oxide; FEV, forced expiratory volume in 1 s; FVC, forced vital capacity; IQR, interquartile range; n, number of patients; PC20M, provocative methacholine concentration causing a fall in FEV1 of 20%.

### 3.2 Correlation between asthma‐related quality of life and demographic and disease characteristics

The correlation between demographic and clinical parameters and the AQLQ variable and its four subscales are reported in Table 3. ACT was strongly positively correlated with global AQLQ score (*r* = 0.81) and its four subscales. To a lesser extent, FEV_1_ pre (%), FEV_1_ post (%), FVC pre (%), and FVC post (%) were positively correlated with global AQLQ score and its four subscales. BMI was weakly and inversely correlated with global AQLQ score and its symptoms and activity subscales. All of the inflammatory parameters were inversely correlated with global AQLQ score except for FeNO and total serum IgE, which displayed no correlation. Those two inflammatory parameters did, however, show a significant relationship with two of the subscales: FeNO was positively associated with the activity dimension and total serum IgE was negatively associated with the emotional dimension. Among the inflammatory parameters, blood neutrophils exhibited the strongest relationships.

**TABLE 3:** Correlation between AQLQ scores and demographic and disease characteristics

	Global AQLQ		Symptom dimension		Activity dimension		Emotional dimension		Environmental dimension	
	Correlation	*p*‐value	Correlation	*p*‐value	Correlation	*p*‐value	Correlation	*p*‐value	Correlation	*p*‐value
ACT	0.81	<0.0001	0.79	<0.0001	0.76	<0.0001	0.71	<0.0001	0.48	<0.0001
Age (years)	−0.01	0.84	0.04	0.12	−0.12	<0.0001	0.01	0.64	0.04	0.11
Age of onset of asthma (years)	0.06	0.04	0.04	0.26	−0.03	0.31	0.10	0.001	0.14	<0.0001
BMI (kg/m^2^)	−0.12	<0.0001	−0.10	0.0003	−0.21	<0.0001	−0.07	0.009	−0.03	0.26
FEV_1_ pre (% pred.)	0.31	<0.0001	0.26	<0.0001	0.34	<0.0001	0.29	<0.0001	0.15	<0.0001
FEV_1_ post (% pred.)	0.31	<0.0001	0.26	<0.0001	0.34	<0.0001	0.28	<0.0001	0.15	<0.0001
FVC pre (% pred.)	0.28	<0.0001	0.22	<0.0001	0.34	<0.0001	0.23	<0.0001	0.13	<0.0001
FVC post (% pred.)	0.24	<0.0001	0.18	<0.0001	0.30	<0.0001	0.19	<0.0001	0.12	<0.0001
FEV_1_/FVC pre (%)	0.12	<0.0001	0.10	<0.0001	0.11	<0.0001	0.16	<0.0001	0.02	0.51
FEV_1_/FVC post (%)	0.14	<0.0001	0.13	<0.0001	0.15	<0.0001	0.17	<0.0001	0.03	0.36
FeNO (ppb)	0.04	0.13	0.03	0.25	0.09	0.0007	−0.02	0.47	−0.01	0.84
Fibrinogen (g/L)	−0.14	<0.0001	−0.12	<0.0001	−0.19	<0.0001	−0.08	0.003	−0.06	0.04
CRP (mg/L)	−0.10	0.0004	−0.11	<0.0001	−0.14	<0.0001	−0.04	0.17	−0.03	0.22
Total serum IgE (kU/L)	0.00	0.98	0.01	0.65	0.03	0.24	−0.08	0.006	0.00	0.93
Sputum neutrophils (10^3^/g)	−0.06	0.04	−0.06	0.07	−0.08	0.01	−0.03	0.37	−0.05	0.14
Sputum eosinophils (10^3^/g)	−0.09	0.006	−0.09	0.004	−0.06	0.08	−0.10	0.002	−0.06	0.05
Blood neutrophils (1/μl)	−0.20	<0.0001	−0.19	<0.0001	−0.22	<0.0001	−0.17	<0.0001	−0.07	0.01
Blood eosinophils (1/μl)	−0.08	0.004	−0.08	0.007	−0.07	0.01	−0.10	<0.0001	−0.04	0.12

*Note*: Correlation was calculated using the Spearman coefficient.

Abbreviations: ACT, Asthma Control Test; AOLQ, Asthma Quality of Life Questionnaire; BMI, Body Mass Index; CRP, C‐reactive protein; FENO, fraction of exhaled nitric oxide; FEV, forced expiratory volume in 1 s; FVC, forced vital capacity.

### 3.3 Multivariate beta regression

The results of multivariate beta regression analysis are presented in Table 4. ACT was the only determinant displaying significant association with global AQLQ (*p* < 0.0001) and its four subscales (*p* < 0.0001). None of the other independent variables was associated with global AQLQ. Gender had an influence on the activity and environmental domains; females had lower asthma‐related quality of life scores in the activity (*p* = 0.047) and environmental (*p* = 0.013) domains than did males. Likewise, treatment had a significant association with the emotional and environmental subscale scores. That is, patients being treated with ICS + LABA and/or LTRA and/or LAMA and patients receiving any maintenance treatment + OCS had lower scores on the asthma‐related quality of life emotional and environmental subscales than did untreated patients (*p* < 0.05). Smokers had higher asthma‐related quality of life scores for the environmental subscale than did non‐smoking patients (*p* < 0.0001). BMI was inversely associated with the activity subscale (*p* < 0.0001), while FeNO (ppb) was positively associated with this subscale (*p* < 0.0001). Post‐bronchodilation FVC and sputum neutrophils were inversely associated with the symptom dimension of AQLQ (*p* < 0.05), while the reverse was found for post‐bronchodilation FEV1 (*p* < 0.05).

**TABLE 4:** Multivariate beta regression results for asthma‐related quality of life and its four dimensions

			Global AQLQ		Symptom dimension		Activity dimension		Emotional dimension		Environmental dimension	
			Estimate	*p*‐value	Estimate	*p*‐value	Estimate	*p*‐value	Estimate	*p*‐value	Estimate	*p*‐value
ACT			0.139**	<0.0001	0.153**	<0.0001	0.157**	<0.0001	0.144**	<0.0001	0.086**	<0.0001
Gender (male)			0.055	0.265	0.0006	0.992	0.115*	0.047	−0.1116	0.156	0.207*	0.013
Age (years)			−0.0001	0.978	0.0004	0.871	−0.0026	0.334	0.0011	0.733	−0.00016	0.963
Smoking status		Ex‐smokers	0.609	0.457	−0.0038	0.953	−0.0428	0.575	−0.0295	0.748	−0.0402	0.679
		Current smokers	0.161	0.082	−0.0951	0.174	0.0654	0.426	0.1079	0.279	0.411**	<0.0001
Atopy (yes)			0.0410	0.445	0.1026	0.093	0.0683	0.338	−0.0105	0.902	−0.0076	0.933
Age of onset of asthma (years)			0.0002	0.909	−0.0003	0.873	−0.0029	0.136	0.000009	0.996	0.0039	0.117
Fibrinogen (g/L)			−0.0060	0.855	−0.0002	0.996	−0.0226	0.604	−0.0238	0.651	0.0409	0.461
CRP (mg/L)			0.0019	0.509	−0.0012	0.706	−0.0004	0.903	0.007	0.097	0.0014	0.764
Total IgE (KU/L)			−0.000004	0.891	−0.000005	0.897	0.00004	0.257	−0.00003	0.649	−0.00004	0.454
FEV_1_ pre (%)			0.0006	0.904	−0.0063	0.253	−0.0016	0.807	0.0116	0.133	−0.0025	0.755
FEV_1_ post (%)			0.0046	0.354	0.0119*	0.035	0.0085	0.202	−0.0021	0.788	−0.0011	0.895
FVC pre (%)			0.0021	0.695	0.0088	0.145	0.0081	0.247	−0.0079	0.352	−0.0005	0.951
FVC post (%)			−0.0029	0.588	−0.0119*	0.048	−0.0068	0.333	0.0039	0.637	0.0047	0.601
Sputum neutrophils (10^3^/g)			−0.000004	0.263	−0.000007*	0.045	−0.000007	0.114	0.0000004	0.942	−0.000002	0.831
Sputum eosinophils (10^3^/g)			0.000007	0.486	0.00001	0.285	0.000004	0.787	0.000024	0.176	0.000003	0.861
Blood eosinophils (μl)			0.00003	0.549	−0.00002	0.765	−0.000022	0.764	0.00011	0.218	0.0001	0.257
Blood neutrophils (μl)			−0.000003	0.606	0.000001	0.827	0.000003	0.666	−0.000008	0.323	−0.00001	0.294
FeNO (ppb)			0.0009	0.156	−0.0004	0.574	0.0033**	<0.0001	−0.000035	0.971	0.00002	0.981
BMI (kg/m^2^)			−0.0049	0.310	−0.0021	0.704	−0.0212**	<0.0001	0.0037	0.637	0.0049	0.550
Exacerbations (yes)			−0.0193	0.729	−0.0139	0.827	−0.0262	0.724	−0.0585	0.513	0.0492	0.603
Asthma treatment	SABA alone as needed		−0.0117	0.895	0.0544	0.580	0.0248	0.834	−0.0909	0.517	−0.0318	0.828
	ICS + LABA and/or LTRA and/or LAMA		−0.0929	0.270	0.0802	0.391	−0.0374	0.739	**−0.3559** ^*^	0.007	**−0.275** ^*^	0.047
	Maintenance treatment + OCS		−0.1764	0.125	0.0618	0.634	−0.01447	0.345	−0.4833*	0.008	−0.439*	0.022
Akaike Information Criterion			−880.0074		−721.6109		−650.7836		−440.0795		−245.8956	

Abbreviations: ACT, Asthma Control Test; AOLQ, Asthma Quality of Life Questionnaire; BMI, Body Mass Index; CRP, C‐reactive protein; FENO, fraction of exhaled nitric oxide; FEV, forced expiratory volume in 1 s; FVC, forced vital capacity; ICS, inhaled corticosteroids; LABA, long‐acting beta agonists; LAMA, long‐acting antimuscarinics; LTRA, leukotriene receptor antagonists; OCS, oral corticosteroids; SABA, short acting beta agonists.

*Significant at the *p* < 0.05 level. ** In bold: significant at the *p* < 0.0001 level.

### 3.4 Relationship between FeNO and the AQLQ activity dimension

**FIGURE 3:**
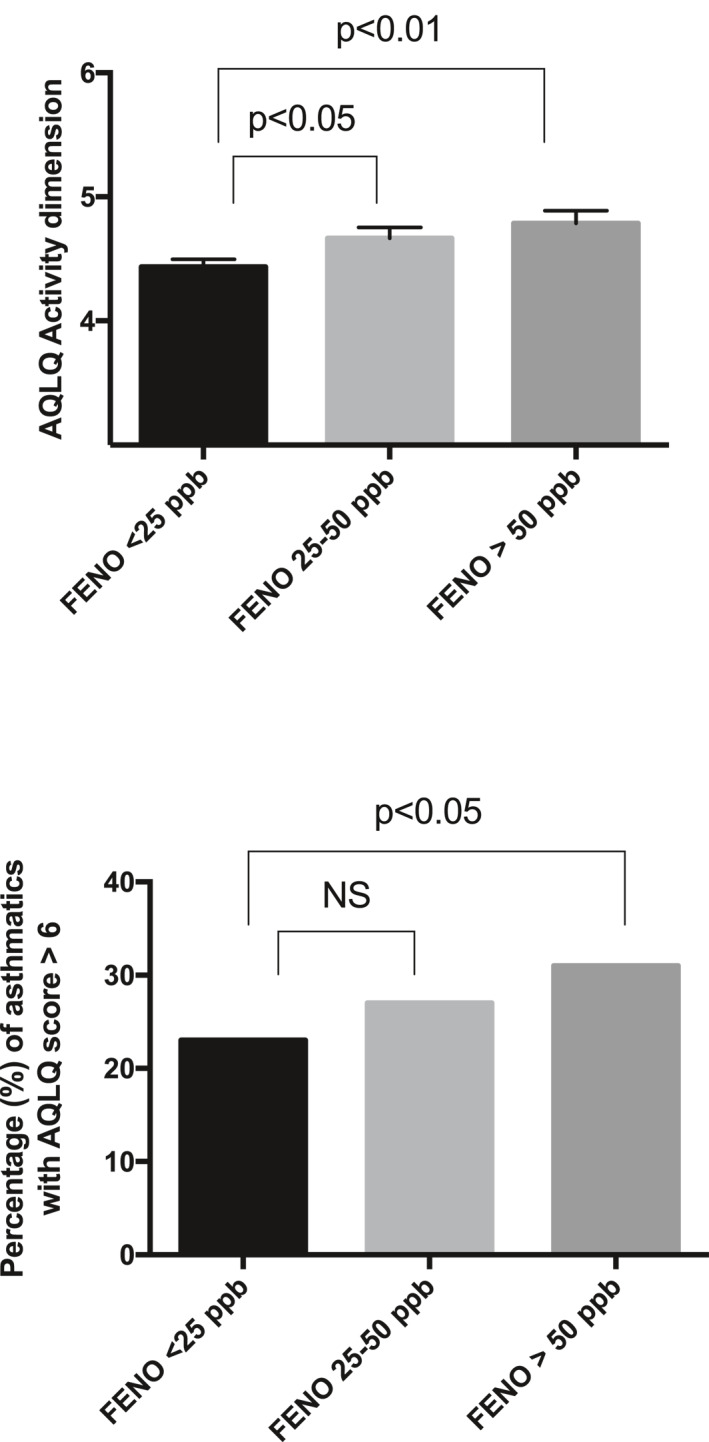
Comparison of the asthma‐related quality of life (AQLQ) activity dimension between patients grouped according to the American Thoracic Society classification for FeNO values (upper panel) and proportion of patients with high AQLQ (AQLQ ≥ 6) in the activity dimension (lower panel). Results are expressed as mean ± standard error of the mean. FeNO < 25 ppb group (*N* = 698), FeNO 25–50 ppb group (*N* = 329), and FeNO > 50 ppb group (*N* = 252) (upper panel)

When we applied the FeNO value categories recommended by the American Thoracic Society clinical practice guidelines, we found that the group with low FeNO values (<25 ppb) had the poorest reported quality of life (Figure 3, upper panel). There was a gradual increase in the proportion of patients reporting high AQLQ (≥6), from 23% in the low FeNO group to 31% in the high FeNO group (OR = 0.66; 95 CI from 0.48 to 0.91; *p* < 0,05) (Figure 3, lower panel).

## 4 DISCUSSION

In this large cross‐sectional study, we identified several predictors of asthma‐related quality of life and/or its four dimensions as measured by the AQLQ. First, our results show that ACT is the factor most significantly associated with global AQLQ and its four dimensions (symptoms, activity limitation, emotional function, and environmental stimuli). Second, our results show that some demographic characteristics—gender, BMI, smoking status, and treatment—are predictors of certain dimensions of asthma‐related quality of life. Third, while cellular inflammatory and lung function parameters are correlated to the global AQLQ, the correlation no longer holds true after multivariate analysis. Finally, and this is the most original finding of our study, FeNO appears to be positively associated with the activity dimension.

### 4.1 Relationship between asthma‐related quality of life and asthma control

The level of asthma control as assessed by ACT was identified as the leading predictor of asthma‐related quality of life as measured by the AQLQ. Indeed, it was the only factor strongly associated with global AQLQ and all of the AQLQ dimensions. These results are in line with previous studies indicating that asthma control had a strong influence on HRQL.^7^, ^8^, ^9^, ^26^, ^27^, ^28^, ^29^ Chen et al.^8^ using a multivariate analysis in a large cohort, found an inverse relationship between asthma control (measured by Asthma Therapy Assessment Questionnaire) and both asthma‐specific HRQL, measured by the Mini AQLQ, and generic HRQL, measured by EuroQol 5‐D. Likewise, Pereira et al. (2011)^26^ reported an inverse relationship between asthma control (measured by ACT) and asthma‐specific HRQL as measured by the Saint Georges's Respiratory Questionnaire (SGRQ), but that study investigated only a limited number of patients. The strong relationship between ACT and AQLQ in our study (rs = 0.81) can be explained, in part, by the fact that two of the AQLQ's dimensions (activity limitation and symptoms) are also found in the ACT. The overlap of common domains is likely to strengthen the relationship between the two questionnaires.^28^

### 4.2 Relationship between asthma‐related quality of life and demographics

In our study, although there was no difference in global AQLQ, women reported poorer asthma‐related quality of life in the activity limitation and environmental stimuli domains. Our finding of a significant association between gender and the AQLQ dimensions of activity limitation and environmental stimuli is perfectly in line with the study by Naleway et al.^10^ Other studies reported that women with asthma experienced a lower HRQL^11^, ^13^, ^30^, ^31^ Some authors^30^, ^32^ believe that the relationship between gender and asthma‐related quality of life is related to the higher prevalence of anxiety and depression among women compared to men. While our study found no gender differences in the AQLQ's emotional dimension, that dimension does not take anxiety and depression into account.

An earlier study reported that asthmatic smokers had a lower generic HRQL.^11^ We were unable to confirm this finding when focusing solely on asthma‐related quality of life. On the contrary, in the present study smokers had a higher asthma‐related quality of life in the environmental domain than did non‐smokers. Though this seems surprising, it can be explained by the composition and formulation of the AQLQ's environmental questions. Of the three questions that make up the AQLQ's environmental domain, one asks whether the patient has been bothered by cigarette smoke. It is very likely that most smokers responded “rarely or never” to this question. It is worth noting, moreover, that the relationship between smoking and asthma‐related quality of life is different than that between smoking and asthma control, where smoking has a clear detrimental effect on the level of control.^33^ This illustrates the fact that although asthma control and asthma‐related quality of life are strongly related, they reflect different dimensions of the disease.

Obesity is a major comorbidity in asthma, affecting up to 25%–50% of severe asthmatics, depending on the country.^34^ In our study, BMI was negatively, though weakly, associated with the AQLQ activity limitation subscore. This means that the higher the asthma patient's BMI, the poorer his or her asthma‐related quality of life in the activity dimension. This finding is consistent with another study by Lavoie et al.^14^ and is readily understandable given the burden obesity places on everyday movement. It also demonstrates the importance of considering comorbidities when assessing asthmatics' quality of life.

Our study showed no association between either atopy—which was present in more than half of the asthma cohort—or total serum IgE and global asthma‐related quality of life or any of its subscales. We therefore could not confirm that non‐atopic asthma might have a greater negative impact of asthma‐related quality of life, as found in a previous study.^35^ In that study, over 70% of the cohort were atopic patients who were younger and had less severe disease; only half were being treated with ICS and the average FEV_1_ was greater than 90% predicted. In our cohort, atopic patients had an average baseline FEV_1_ of 84% predicted, and 59% were being treated with ICS/LABA (data not shown). We therefore believe that our population of atopic asthmatics had more severe disease than that of Ek et al.^35^ Our data support the hypothesis that atopy is a prominent trait associated with asthma, but is not a determinant factor in worsening or improving quality of life.

Our data indicate that treatment burden has an impact on the emotional and environmental dimensions of asthma‐related quality of life. Unlike patients who receive SABA alone as needed, the patients receiving maintenance treatment (ICS/LABA and/or LTRA and/or LAMA)—and those receiving OCS, in particular—showed diminished quality of life. Presumably, the intensity of the pharmacological treatment makes the patients more aware of their fragility in anxiety‐producing and stressful situations.

### 4.3 Relationship between asthma‐related quality of life and lung function

Confirming previous results,^8^, ^13^, ^15^ we found a low inverse relationship between FEV_1_ and global AQLQ, supporting the idea that airflow limitation results in impaired quality of life. This relationship remained significant after multiple regression analysis, but only for the symptom dimension of the AQLQ. This supports the hypothesis that FEV_1_ contributes to the scoring of the symptom dimension of asthma‐related quality of life.

### 4.4 Relationship between asthma‐related quality of life and inflammation

Airway inflammation is an essential component of asthma. To the best of our knowledge, no studies have looked at the direct relationship between asthma‐related quality of life and airway or systemic inflammation. Thus, one unique aspect of our study was its exploration of the link between asthma‐related quality of life and airway inflammation. The magnitude of neutrophilic and eosinophilic inflammation both at the systemic and airway level correlates inversely with global AQLQ score; the more eosinophils and/or neutrophils in the sputum and blood, the poorer the asthma‐related quality of life. Only the association between sputum neutrophils and the symptom dimension of AQLQ remained significant after multiple regression analysis. The fact that the relationship between sputum eosinophils and AQLQ disappeared after multiple regression analysis suggests that the observed correlation between sputum eosinophils and AQLQ was mediated through poor asthma control. This is consistent with previous studies—both cross‐sectional and longitudinal—that have shown that sputum eosinophils are independent determinants of the fluctuation in asthma control.^33^, ^36^, ^37^

Finally, our multivariate analysis yielded a surprising and interesting result regarding FeNO. It showed that FeNO was positively associated with the activity dimension of asthma‐related quality of life. High FeNO has traditionally been seen as a bad outcome reflecting intense eosinophilic inflammation.^38^ In addition, Roberts et al.^17^ focusing on teenagers with allergic asthma, demonstrated that quality of life declines when FeNO increases as a result of pollen allergen exposure. In contrast, Ehrs et al.^16^ found no relationship between FeNO and AQLQ in a population of mild, untreated asthmatics. Caminati et al.^18^ showed that AQLQ scores in patients with severe allergic asthma were lower in patients with FeNO ≥30 ppb than in patients with FeNO <30 ppb. None of these studies used multiple regression analysis, and FeNO may be affected by other factors likely to impact its effect on AQLQ in a univariate analysis, such as airway eosinophilia. We therefore believe that our statistical methods better reflect the true association between FeNO and quality of life. The reason why FeNO might positively affect the activity dimension of quality of life remains to be investigated, but it is worth noting that NO is a recognized mediator of vasodilation,^39^ which is a physiological process of utmost importance in physical activity. While FeNO is usually considered to be the consequence of activation of inducible NO synthase due to local inflammation,^40^ we cannot rule out the possibility that part of FeNO actually reflects an inherited propensity of the body to synthesize NO.

### 4.5 Limitations of the study

The strength of this study lies in the size of the cohort, the use of validated PROMs (Mini AQLQ and ACT), and the inclusion of clinically well‐characterized asthma patients; our study does, however, have several limitations. As this was a cross‐sectional study, the cause and effect of these associations cannot be determined. Another limitation is the lack of sociodemographic data such as the occupation or education level, which are likely to influence asthma‐related quality of life.^3^, ^8^ The strength of the relationship between asthma control and asthma‐related quality of life might have been weaker, if asthma‐related quality of life had been assessed by a different questionnaire that considered other quality‐of‐life dimensions.^41^ Finally, our analysis did not include several comorbidities—such as rhinosinusitis or gastroesophageal reflux—known to impact asthma patients' quality of life.^35^, ^42^, ^43^

## 5 CONCLUSION

Asthma control is the main predictor of asthma‐related quality of life and impacts all of its dimensions, but demographic, functional, and airway inflammatory parameters may also influence some aspects of asthma‐related quality of life. Among the airway inflammatory parameters, FeNO emerged as the inflammatory factor most significantly associated with AQLQ, and surprisingly the relationship between FENO and the activity dimension was positive. Whatever the reason for this, our study shows that considering patient‐reported outcomes might refine clinicians' views on an objective physiological parameter.

## CONFLICT OF INTEREST

Outside of this submitted work, RL received unrestricted research grants from GSK, AstraZeneca, Novartis and Chiesi and lecture or adboard fees from GSK, AZ, Novartis and Sanofi. Outside of this submitted work, FS received lecture or adboard fees from Chiesi, AZ, GSK, and Novartis. The remaining authors declare that they have no relevant conflicts of interest.

## AUTHOR CONTRIBUTIONS

GL, BP, FS and RL contributed to the design of the study. FS, FG, MH, VP and RL contributed to data acquisition. GL, HNZ, AFD performed the statistical analysis. GL, BP, FS, RL, MG and AS drafted and critically revised the work. All authors gave final approval of the manuscript.

## CONSENT FOR PUBLICATION

Not applicable.
